# Hungry Hematopoietic Stem Cells during Bacterial Infection: Fatty Acid for Food

**DOI:** 10.20900/immunometab20220011

**Published:** 2022-04-02

**Authors:** Marie-Dominique Filippi

**Affiliations:** Division of Experimental Hematology and Cancer Biology, Cincinnati Children’s Hospital Research Foundation, Department of Pediatrics, University of Cincinnati College of Medicine, Cincinnati OH 45229, USA

**Keywords:** hematopoietic stem cells, infection, fatty acid, CD36, mitochondria

## Abstract

Hematopoietic stem cells (HSC) directly initiate a response to bacterial infections by rapidly entering the cell cycle in order to produce mature blood cells. An important issue in the field of HSC biology is to understand how metabolic activities of HSC are fueled during specific condition that require HSC activation. In their paper, Mistry et al. provide evidence that bacterial infections trigger an increased in free fatty acid uptake by HSC that fuel fatty acid oxidation and mitochondrial respiration activities. This increased fatty acid uptake is exclusively dependent on the upregulation of the fatty acid transporter CD36. This study shed important light into the metabolic needs of HSC during septic conditions.

Hematopoietic stem cells rapidly respond to infection in order to meet the demand in mature blood cell production, known as emergency hematopoiesis [1]. Whether it is due to direct pathogen recognition or response to inflammatory cytokines that are released by other cells, normally quiescent HSC initiate a response to infection by entering the cell cycle, proliferating and differentiating. The transition from quiescence to activation is inherently accompanied by major metabolic changes. Quiescent HSC have low mitochondrial respiratory activity, albeit keeping high mitochondrial content, and rely on anaerobic glycolysis and fatty acid oxidation (FAO) [2,3]. Other pathways are important as well, such as non-canonical retinoic acid signaling and autophagy [4–6]. An exit from quiescence on the other hand requires increased mitochondrial oxidative phosphorylation, increased glycolysis and FAO, as well as the activation of purine metabolism and aspartate availability [5,7–9]. Yet, our knowledge of the metabolic needs of HSC is in its infancy—owing to the difficult metabolic profiling of these rare cells with classical mass spectrometry approaches. Much remains to be learnt on how HSC metabolic activity is fueled and on the context specificity of metabolite utilization.

Fatty acid oxidation is a cyclical reaction to break down fatty acids (2 carbons by cycle) into acetyl-CoA. FAO occurs mainly in the mitochondria or into peroxisomes for very long chains FA. FAs are taken up by cells via plasma membrane transporters, are catalyzed into fatty acyl-CoA molecules and then transferred into mitochondria via the mitochondrial carnitine palmitoyltransferase I (CPT1). Acetyl-CoA generated from FAO can enter the tricarboxylic acid (TCA) cycle to fuel oxidative phosphorylation coupled with energy production [10]. FAO is critically important for HSC maintenance and for the differentiation of their progeny. FAO can be utilized both to maintain HSC quiescence and for HSC self-renewal during HSC expansion ex vivo [7,11]. While the importance of FAO for HSC homeostasis is established, how FA enter HSC is largely unknown. Equally importantly, how HSC use FAO during emergency hematopoiesis is unknown.

In their recent publication, Mistry et al. show that uptake of free FA (FFA) by HSC drastically increases after *S. typhimurium* infection—hence, leading to an increase in FAO, OXPHOS and energy production [12]. They used a luciferase reporter system to measure real-time FFA uptake in vivo as well as BODIPY-dodecanoic acid fluorescent probe, allowing them to show that HSC and multipotent progenitors (HSPC) all acquire FFA in response to bacterial infection in vivo. There are several ways for a cell to take up FFA, including CD206, CD36, fatty-acid-binding proteins and fatty-acid transport proteins. Quite interestingly, they identify CD36 as the specific FFA transporter responsible for increased FFA uptake during infection. Only CD36 expression was upregulated in response to LPS or infection in HSPC. CD36 is a class B scavenger receptor that bind multiple ligands, including oxidized LDL, fatty acids, collagen, thrombospondin, and anionic phospholipids. It is also known as platelet glycoprotein 4, and is highly expressed in erythroid progenitors. Mistry et al. used elegant orthogonal approaches to demonstrate the functional importance of CD36 and FAO in HSC response to infections, including a specific inhibitor of CD36 (sulfosuccinimidyl), the inhibitor of FAO etomoxir, and hematopoietic progenitors expressing a *cpt1a*-knock-down construct. In each case, blocking CD36-driven FFA uptake and subsequent FAO in hematopoietic progenitors blocked HSPC expansion and emergency hematopoiesis during infection. As a result, mice were more susceptible to liver injury and succumbed to infection. Reciprocal mouse transplant experiments using genetic CD36-deficiency confirmed the HSPC cell autonomous nature of this pathway [12]. Hence, HSCs have increased reliance on FAO for their expansion in response to bacterial infection (Figure 1).

Interestingly, CD36 seems specifically needed for FFA uptake by HSC in response to infection but not in other stress conditions that still rely on FAO. CD36 is not expressed in HSC at steady state, and CD36-deficiency does not alter HSC repopulation potential in non-infection related context such as classical transplant studies. Not only do CD36^−/−^ HSCs possess full regenerative potential, CD36^−/−^ HSCs also produce a balanced myeloid-lymphoid graft, indicating that CD36-driven FFA uptake in HSCs is a specific response to LPS and bacterial infection [12]. Broadly, CD36 is also critically important for tumor development. During blast crisis chronic myeloid leukemia, CD36 expression segregates leukemic stem cells (LSCs) into 2 metabolically and functionally distinct populations. CD36-positive LSCs have high FAO activity, have a quiescent phenotype and are poised to resist chemotherapy in adipose-enriched environment [13]. Together, these findings are important as it means that CD36 plays a role in HSPC functions in a very specific context-dependent manner. CD36-mediated FFA uptake may offer a therapeutic window for use in emergency condition related to infections. As noted above, CD36 can also bind other ligands, the functions of which during infections is currently unknown and will be important to examine.

The source of FFA in the bone marrow during infection is unknown. In the case of leukemia, the adipose tissue is highly lipolytic releasing FFA to be taken up by LSCs—thus generating a pro-tumoral microenvironment [13,14]. As noted by the authors, the bone marrow environment is rich in adipocytes, which normally represent a biologically active energy storage for maintaining HSC homeostasis. In the context of infection, adipocytes may also be the source of FFA, but perhaps indirectly via the inflammatory cytokine, interleukin 6 (IL6) [12]. Indeed, IL6 can control the uptake and release of FAs from adipocytes. It will be important to examine this further and identify exactly what fuels HSC FAO during infection as well as in other contexts. In addition, how FFA are used beyond oxidation in the mitochondria will need to be explored. FFA can be incorporated into cellular membranes modifying their organization and cooperating with membrane cholesterol. Most notably, cholesterol efflux via a different class of lipid transporters, Abca1 and Abcg1, is critically important for HSPC proliferation during atherosclerosis. In this context, a change in cholesterol membrane composition is needed for the formation of cholesterol-rich lipid rafts in order to facilitate cytokine signaling [15]. A link between FFA and cholesterol in the context of HSPC proliferative response to infections will be important to examine.

This study brings into focus several important questions: how a change in fatty bone marrow environment and nutrient availability impact HSC response to bacterial infections, what happens during chronological aging, which is accompanied by an increase in adipocyte content, or during obesity and metabolic disorders? How do changes in FFA availability reprogram HSC metabolism? Equally important will be to understand what fuels FAO, what is the source of FFA, and how HSC take up FFAs to maintain HSC functions at steady state or under non-infection related regenerative conditions. Answering these questions is critical to exploit the metabolic needs of HSC during emergency hematopoiesis for clinical purposes.

## Figures and Tables

**Figure 1.**
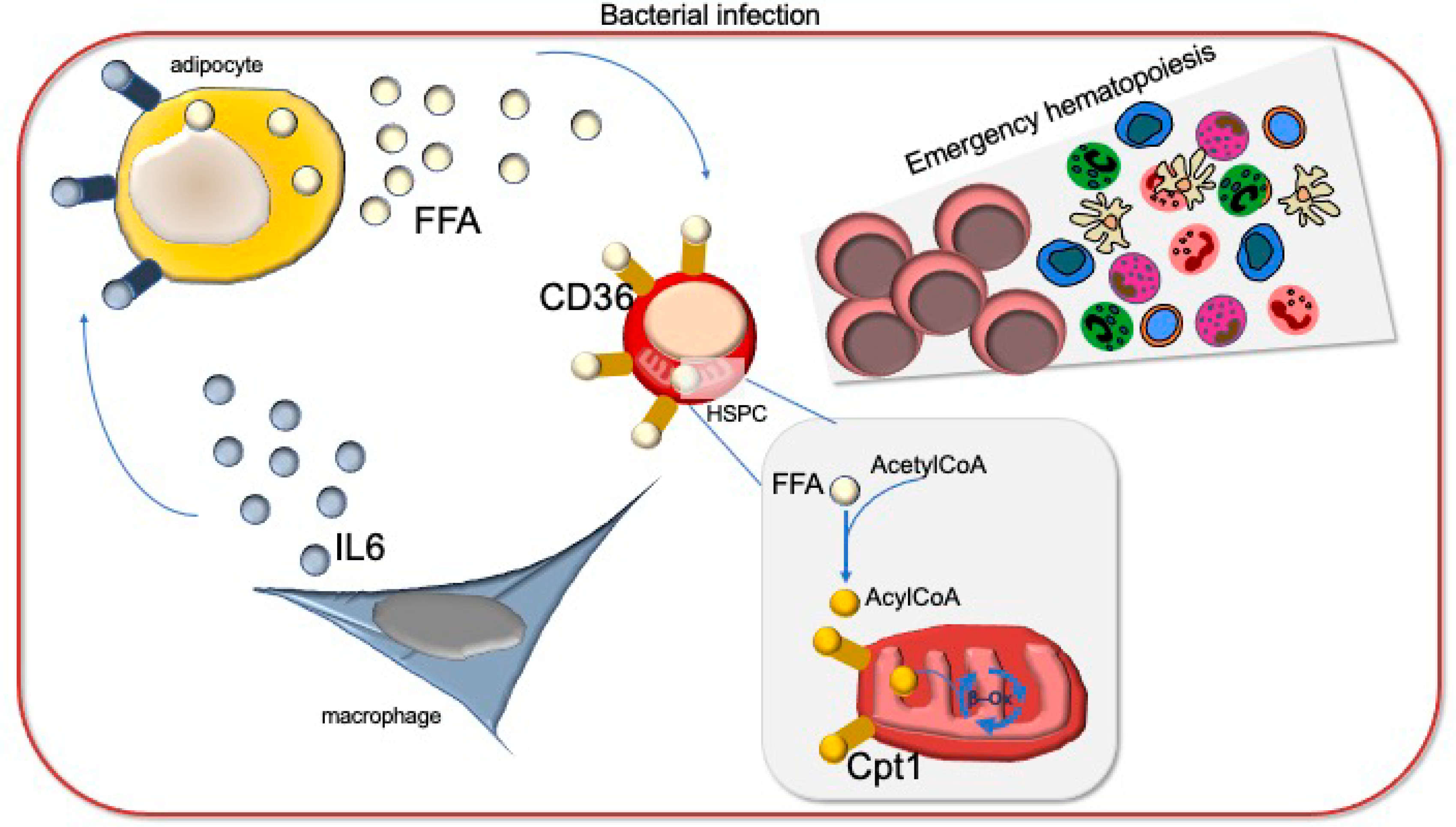
HSPC response to bacterial infection. Following bacterial infection, hematopoietic stem and progenitor cells upregulate CD36 expression and take up free fatty acid (FFA) that are likely released in the bone marrow by adipocytes in response to inflammatory cytokines IL6. As a result, HSCs rely on fatty oxidation for their energy demand to expand the pool of HSPC and produce mature blood cells in order to protect the body against the infection.
